# Exploring the influence of lactoferrin on wound healing and related signaling pathways: a systematic review

**DOI:** 10.1002/2211-5463.70298

**Published:** 2026-09-09

**Authors:** Morgana Lüdtke Azevedo, Kátia Cristiane Hall, Matheus dos Santos Fernandez, Geovanna Peter Corrêa, Rafael Guerra Lund

**Affiliations:** ^1^ Graduate Program in Biochemistry and Bioprospecting (PPGBBio) Federal University of Pelotas Pelotas RS Brazil; ^2^ Graduate Program in Dentistry (PPGO) Federal University of Pelotas Pelotas RS Brazil; ^3^ Graduate Program in Materials Science and Engineering (PPGCEM) Federal University of Pelotas Pelotas RS Brazil

**Keywords:** bioprospecting, lactoferrin, regenerative medicine, systematic review, wound healing

## Abstract

This systematic review evaluates the therapeutic efficacy and biological mechanisms of Lactoferrin (Lf) in the wound healing process. The objective was to synthesize evidence regarding its multifunctional role in cellular regeneration, gene modulation, and antimicrobial protection. From an initial screening of 1846 records, 11 *in vitro* studies (2001–2023) met the eligibility criteria. The synthesized data demonstrate that Lf significantly accelerates the migration and proliferation of human keratinocytes and fibroblasts. At the molecular level, Lf upregulates critical regenerative genes, specifically *HAS2* (hyaluronan synthesis) and *COL1A1* (collagen type I). Furthermore, Lf exhibits potent inhibitory effects against biofilm formation and bacterial growth, with high efficacy reported against *Staphylococcus aureus* and *Escherichia coli*. Thus, Lf acts as a pivotal bioactive agent that bridges regenerative stimulus with antimicrobial defense across all healing phases. Its ability to modulate the extracellular matrix and suppress pathogens suggests it to be a candidate for advanced wound care and regenerative therapies. These findings establish a robust biological rationale for transitioning toward clinical‐stage bioprospecting.

## Graphical abstract

Lactoferrin promotes wound healing by activating MAPK, PI3K/Akt, and extracellular matrix pathways, enhancing fibroblast remodeling, proliferation, and re‐epithelialization. These mechanisms highlight its therapeutic potential in inflammation control, tissue repair, and remodeling.

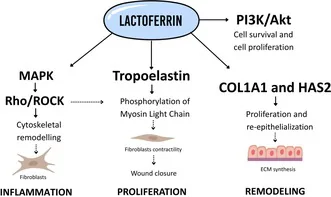

**Abbreviations**
bLf: bovine lactoferrin
COL1A1: collagen, type I; alpha 1
ECM: extracellular matrix
EGF: epidermal growth factor
FGF: fibroblast growth factor
HAS2: hyaluronan synthase
HEKs: human embryonic kidney cells
hLf: human lactoferrin
Lf: lactoferrin
LPS: lipopolysaccharide
LRP1: low‐density lipoprotein receptor‐related protein 1
MLC: myosin light chain
NHDFs: normal human dermal fibroblasts
OSF: Open Science Framework
TE: tropoelastin
TGF‐β: transforming growth factor‐β
VEGF: vascular endothelial growth factor

Wound healing is a highly dynamic and strictly regulated biological process essential for restoring the structural and functional integrity of the skin. It encompasses a coordinated sequence of cellular and molecular events divided into four overlapping phases: hemostasis, inflammation, proliferation and remodeling [1]. Each phase is crucial for successful tissue repair, as disruptions at any stage can lead to healing impairments and abnormal outcomes, such as chronic wounds or excessive scarring (e.g., hypertrophic scars and keloids), which remain significant clinical challenges [2]. This complexity has catalyzed the search for bioactive molecules capable of fine‐tuning the wound‐healing microenvironment to promote effective tissue regeneration [3].

In this context, the bioprospecting of natural compounds has emerged as a cornerstone for developing innovative therapies. Bioactive molecules derived from animal, plant, and microbial sources have gained prominence due to their capacity to modulate key cellular mechanisms [4]. Among these, lactoferrin (Lf) stands out as a particularly interesting candidate because of its multifunctional properties directly related to wound healing. As an iron‐binding glycoprotein, Lf exerts potent antimicrobial, antioxidant, and immunomodulatory effects, which are critical for favorably modulating the wound microenvironment and supporting tissue regeneration [5]. Furthermore, studies have demonstrated its ability to stimulate fibroblast and keratinocyte proliferation, promote angiogenesis, and enhance extracellular matrix (ECM) synthesis, suggesting a potential role in accelerating tissue repair and reducing complications such as infection and chronic inflammation. These characteristics make Lf an attractive molecule for exploration as a therapeutic agent in regenerative medicine [6, 7].

Lf is a member of the transferrin family and is predominantly found in mammalian milk and other exocrine secretions, including saliva, tears, and mucosal fluids. Beyond its classical role in iron homeostasis, Lf's bioactive properties have been widely studied in both human and bovine forms. Both human lactoferrin (hLf) and bovine lactoferrin (bLf) share significant structural similarities, with bLf being widely used in nutraceutical and pharmaceutical applications due to its accessibility and bioactivity [8].

Emerging evidence suggests that Lf contributes to various aspects of wound repair, including acceleration of wound contraction, enhancement of collagen synthesis, and promotion of angiogenesis [9, 10]. Additionally, Lf's capacity to modulate immune responses and mitigate oxidative stress in the wound milieu may further support its therapeutic potential in both acute and chronic wounds [11]. However, differences in Lf's bioactivity related to its source, glycosylation profile, and method of extraction highlight the need for a deeper understanding of its mechanistic roles in tissue repair [12].

Furthermore, despite the promising properties of Lf, the existing literature presents a high degree of heterogeneity regarding experimental models, administration routes, and dosages. Previous reviews have primarily mapped the general availability of studies or focused on isolated aspects of Lf bioactivity. Consequently, there is a lack of a systematic synthesis that critically evaluates the quality of evidence and provides a definitive consensus on the efficacy of Lf across different wound types.

Therefore, a systematic review is necessary to consolidate findings from randomized controlled trials and high‐quality preclinical studies. This study aimed to critically synthesize and evaluate the current evidence on the influence of Lf in wound healing, specifically focusing on its mechanistic efficacy across the four phases of tissue repair. By identifying consistent outcomes and methodological limitations, this review seeks to provide a robust foundation for clinical translation and future pharmacological developments.

## Materials and methods

### Registration and protocol

This systematic review was conducted and reported in accordance with the Preferred Reporting Items for Systematic Reviews and Meta‐Analyses (PRISMA 2020) guidelines. The study protocol was prospectively registered in the Open Science Framework (OSF) (https://osf.io/tsrpc/).

The review aimed to address the following research questions based on the PICO framework:

1. Does Lf administration significantly modulate cellular activities (proliferation, migration, and differentiation) in *in vitro* models of skin wound healing?
2. What are the specific molecular mechanisms by which Lf acts across the different phases of tissue repair?

### Eligibility criteria

The inclusion criteria were restricted to original *in vitro* experimental studies that investigate the influence of Lf on cellular and molecular mechanisms of the wound healing process (e.g., fibroblast and keratinocyte activity, angiogenesis, and inflammatory modulation). The decision to focus exclusively on *in vitro* studies aims to provide a high‐resolution analysis of Lf's direct mechanistic pathways, minimizing systemic confounding factors inherent to *in vivo* models. Exclusion criteria included: (a) secondary studies (reviews, meta‐analyses, editorials, or commentaries); (b) studies using Lf in combination with other active pharmacological compounds where Lf's isolated effect could not be determined; concurrently, functionalized biomaterials, nanocomposites, or molecular associations incorporating Lf were deemed eligible provided that the study design included nonfunctionalized counterparts or matrix controls (such as bare nanoparticles or isolated polymer vehicles), allowing the specific biological contribution and therapeutic added‐value of Lf to be isolated and determined; (c) studies lacking a proper control group; (d) gray literature such as conference abstracts, patents, and book chapters. Only studies published in English were included to ensure the precision of technical‐scientific data extraction. No date restrictions were applied. Google Scholar was not included due to its lower precision for recently emerging topics [13].

### Search strategy

A comprehensive search was performed across PubMed/MEDLINE, Scopus, Web of Science, Cochrane Library, Embase, ScienceDirect, and LILACS. The search included all literature up to January 2026, with no initial date restrictions. The search strategy utilized Medical Subject Headings (MeSH) terms (Table 1).

**Table 1:** Summary of the main characteristics and outcomes of the studies included in this systematic review. This table presents an overview of the studies included in the systematic review, detailing the reference, primary objective, sample size, Lforigin (bovine Lf– bLf; human Lf– hLf), and protocol information (adherence to guidelines for study design and reporting, and whether the protocol was registered in public databases). The main outcomes describe the reported effects of Lf on wound healing and antimicrobial activity in the selected *in vitro* models.

References	Main purpose	Sample (*n*)	Lf origin	Protocol study* (design/reporting/published)	Main outcomes
Abdalla et al., 2020 [6]	To develop nanomaterials with the dual actions of antibacterial and antibiofilm activities using silver nanoparticles (AgNPs) functionalized with either lactoferrin (Lf) or graphene oxide (GO).	*n* = 3	Human Lf	Yes/Yes/No	Chitosan‐stabilized Ag‐Lf demonstrated enhanced antibacterial and antibiofilm activity (Gram‐positive and Gram‐negative) and was nontoxic in cytotoxicity assays.
Abdalla et al., 2021 [38]	To develop gelatin hydrogels incorporated with bio‐nanosilver functionalized with lactoferrin (Ag‐Lf) as a dual‐antimicrobial action dressing, to be used in treating infected wounds.	*n* = 3	Human Lf	Yes/Yes/No	Ag‐Lf‐loaded hydrogel demonstrated antibacterial and antibiofilm activity against selected bacterial strains, was noncytotoxic to HDFs, and did not impair cell migration or gene expression related to normal wound healing.
Belvedere et al., 2021 [7]	To investigate a new association of mesoglycan and Lf, two different substances that, taken alone, have shown notable pro‐healing functions.	*n* = 3	Human milk Lf	Yes/Yes/No	Mesoglycan and Lfexhibited prohealing effects by significantly promoting key molecular processes involved in tissue regeneration during skin wound healing.
Fathil et al., 2023 [10]	To develop a dressing that could effectively aid in the wound healing process and prevent bacterial infections by exerting both antibacterial and antibiofilm effects.	*n* = 3	Human recombinant Lf expressed in rice	Yes/Yes/No	Ag‐Lf demonstrated antibacterial activity against *S. aureus, E. coli*, and *P. aeruginosa*. Ag‐Lf, SL, and DL hydrogels inhibited biofilm formation and enhanced HaCaT cell migration, suggesting pro‐healing effects.
Jiang et al., 2017 [41]	To examine uptake and postpone internalization localization of apo‐ and holo‐Lfs, as well as effects of Lfs and SLs on dermal fibroblasts, and thus to provide preliminary data and a scientific basis for addition of Lf and SLs to cosmetic products.	*n* = 5	Human and bovine Lf isolated from milk, and comercial bovine Lf	Yes/Yes/No	CbLf and CbLf‐SLs assemblies were internalized by dermal fibroblasts and significantly upregulated genes involved in proliferation, ECM synthesis, oxidative stress resistance, and immunomodulation. Both formulations enhanced fibroblast proliferation and migration.
Ryu et al., 2017 [39]	To investigate the molecular mechanisms underlying the increased expression of tropoelastin (TE) mRNA in human dermal fibroblasts induced by bovine Lf.	*n* = 3	Lf, Apo‐Lf, and Holo‐Lf (obtained by iron saturation)	Yes/Yes/No	Lf induced TE expression in dermal fibroblasts, likely via activation of the PI3K/Akt1 signaling pathway, highlighting its potential role in skin elasticity.
Saito et al., 2011 [37]	To study the effects of bovine Lfto see whether it can improve hyaluronan and collagen biosynthesis.	*n* = 3	Bovine Lf	Yes/Yes/No	bLf enhanced hyaluronan synthesis and type I collagen production in NHDFs in a dose‐dependent manner by upregulating HAS2 expression, promoting ECM production and fibroblast motility critical for wound healing.
Takayama et al., 2001 [40]	To report that both human and bovine Lf can enhance collagen gel contraction induced by human fibroblasts, and to elucidate the mechanisms underlying Lf‐induced collagen gel contraction, focusing on the relationship between myosin light chain (MLC) phosphorylation and the gel contraction‐promoting activity of Lf.	Not mentioned.	Purified bLf, purified hLf	Yes/Yes/No	Both bLf and hLf enhanced collagen gel contraction by fibroblasts through Rho/ROCK‐ and MLCK‐dependent pathways, promoting MLC phosphorylation independently of their iron‐binding properties.
Tang et al., 2010 (a) [36]	To test the effects of a transgenic rice‐derived recombinant hLF (holo‐rhLF) on cell proliferation and migration in a primary human skin fibroblast culture system and examined the effects of holo‐rhLF on cell survival and apoptosis.	*n* = 6	Lyophilized holo‐rhLF	Yes/Yes/No	Holo‐rhLf enhanced fibroblast proliferation and migration while protecting cells from apoptosis, highlighting its potential for wound therapy.
Tang et al., 2010 (b) [22]	To evaluate the effects of recombinant human Lf(holo‐rhLF) on skin keratinocyte function and wound re‐epithelialization, both *in vitro* and *in vivo*.	*n* = 6	Lyophilized holo‐rhLF	Yes/Yes/No	Holo‐rhLf promoted keratinocyte proliferation and migration, protected cells from apoptosis, and modulated re‐epithelialization via MAPK, Src, and Rho/ROCK pathways through LRP1 receptor signaling.
Yuan et al., 2021 [9]	To develop and evaluate a novel electrophoresis‐based technology for creating bacterial nanocellulose (BNC) composites functionalized with collagen and Lf, with the aim of improving wound healing applications.	*n* = 3	LF (from cow's milk)	Yes/Yes/No	A BNC/Lf/COL composite membrane developed via electrophoresis exhibited broad‐spectrum antibacterial activity, enhanced biocompatibility and water vapor transmission, and accelerated wound healing, supporting its potential as a clinical wound dressing.

*Note:* According to ISO 10993‐5 standard guidelines for the biological evaluation of medical devices, a material is only classified as cytotoxic if cell viability drops below 70%. In the studies by Abdalla et al. (2020, 2021), although a statistically significant decrease in metabolic activity was noted at higher concentrations compared to controls, cell viability consistently remained above this 70% safety threshold, justifying its classification as non‐cytotoxic.

^*^ Protocol study: indicates whether the study adhered to established guidelines for study design and reporting, and whether the protocol was published in public databases. The information is presented as ‘Yes’ or ‘No’ for each criterion, in the following order: Design/Reporting/Published.

### Study selection and data extraction

Two independent reviewers (MLA and GPC) screened titles and abstracts using Zotero for duplicate removal and initial selection. Full‐text assessment was performed against eligibility criteria. Data extraction was conducted independently by three reviewers (MLA, KCH, and MSF) using a standardized Excel form. Extracted data included: study identification (author, year), Lf source/type, cell lines used, concentrations, treatment duration, and primary outcomes (molecular markers and cellular behavior). Discrepancies were resolved by a third reviewer (RGL).

### Quality and risk of bias assessment

Reliability and quality assessment of all eligible full‐text articles were independently assessed by two reviewers (MLA and KCH) using the ToxRTool (Toxicological Data Reliability Assessment Tool), in its *in vitro* module. This instrument, developed by Schneider and colleagues [14], provides detailed criteria and guidance to evaluate the intrinsic quality of toxicological data, aiming to make the classification of reliability categories more consistent and transparent [15]. Each study was evaluated against 18 predefined binary criteria, and overall scores were used to assign reliability categories based on the Klimisch‐like system [16]. In this framework, Category 1 (‘reliable without restriction’) refers to studies performed in accordance with valid and/or internationally recognized guidelines, preferably under Good Laboratory Practice (GLP) [17]. Category 2 (‘reliable with restrictions’) applies to studies that may not fully adhere to GLP standards but are nevertheless well‐documented and scientifically acceptable. Category 3 (‘not reliable’) is reserved for studies presenting substantial methodological limitations and insufficient documentation; although they may still provide supportive information, their internal validity is compromised. To evaluate inter‐rater reliability, the level of agreement between the two reviewers was quantified using Cronbach's α, calculated from the scores attributed to each study. Any major methodological weaknesses identified were recorded qualitatively as free text and are presented in a summary table within the Results section.

### Data synthesis

A qualitative synthesis was performed to integrate findings regarding Lf's efficacy in wound healing phases. Given the anticipated heterogeneity in cell lines and Lf concentrations, a meta‐analysis was not planned; however, results were grouped by biological mechanism (e.g., anti‐inflammatory, pro‐proliferative, or remodeling) to provide a structured overview of Lf's therapeutic potential.

## Results and discussion

### Study selection and inclusion

**Fig. 1:**
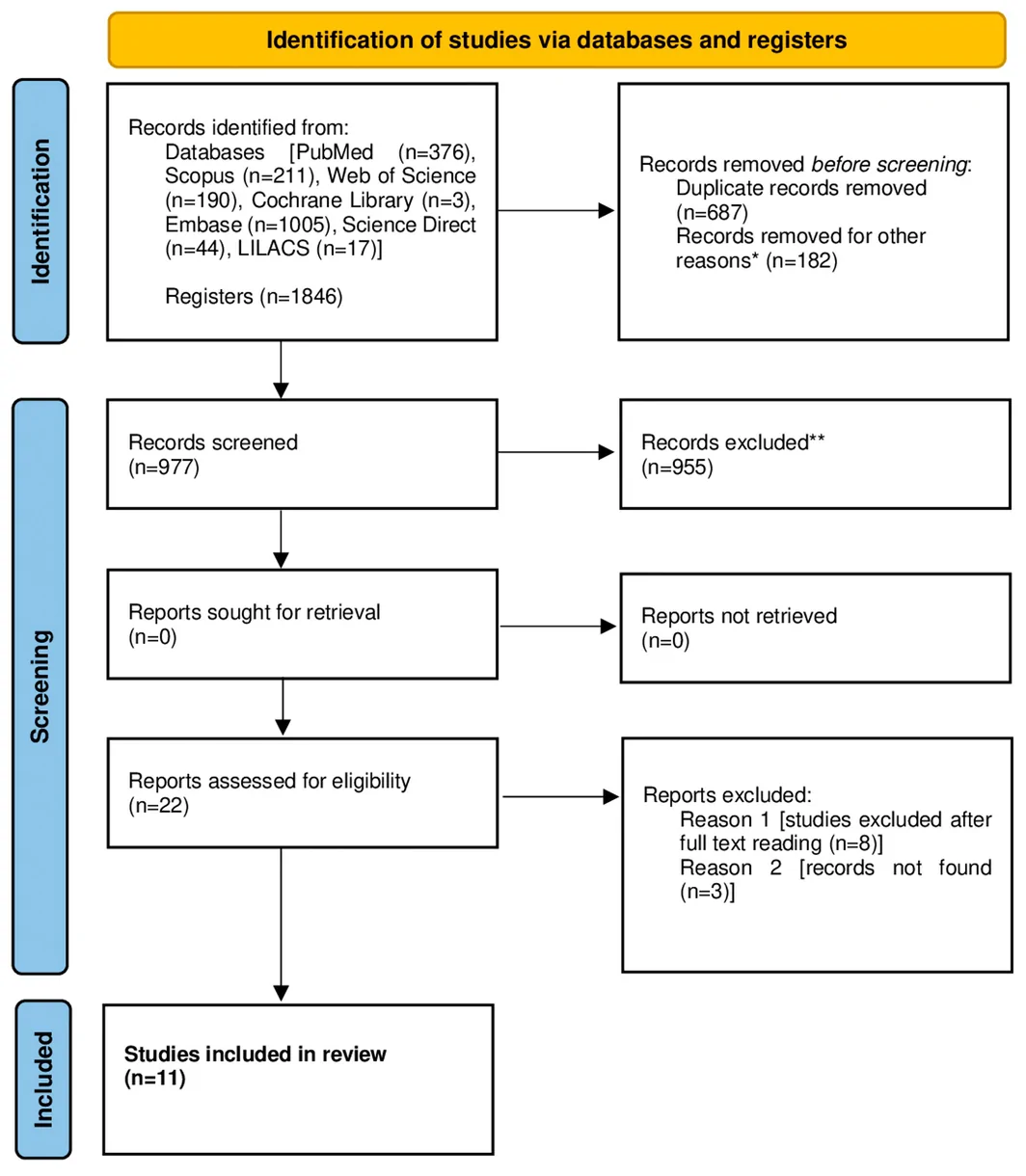
PRISMA 2020 flow diagram for the systematic review. The diagram illustrates the identification of studies via databases and registers. * Records removed for other reasons (*n* = 182): Records excluded by titles and abstracts for not being original articles. ** Records excluded (*n* = 955): Records excluded by titles and abstracts for not analyzing the influence of lactoferrin on wound healing.

The initial search retrieved 1846 records from the selected databases. After removing duplicates, 1159 records remained for title and abstract screening. Of these, 977 articles were selected for full‐text review. Following the full‐text assessment, 955 articles were excluded for not meeting the inclusion criteria. Among the remaining 22 articles, an additional 11 were excluded because they did not perform *in vitro* analyses (Fig. 1).

**Fig. 2:**
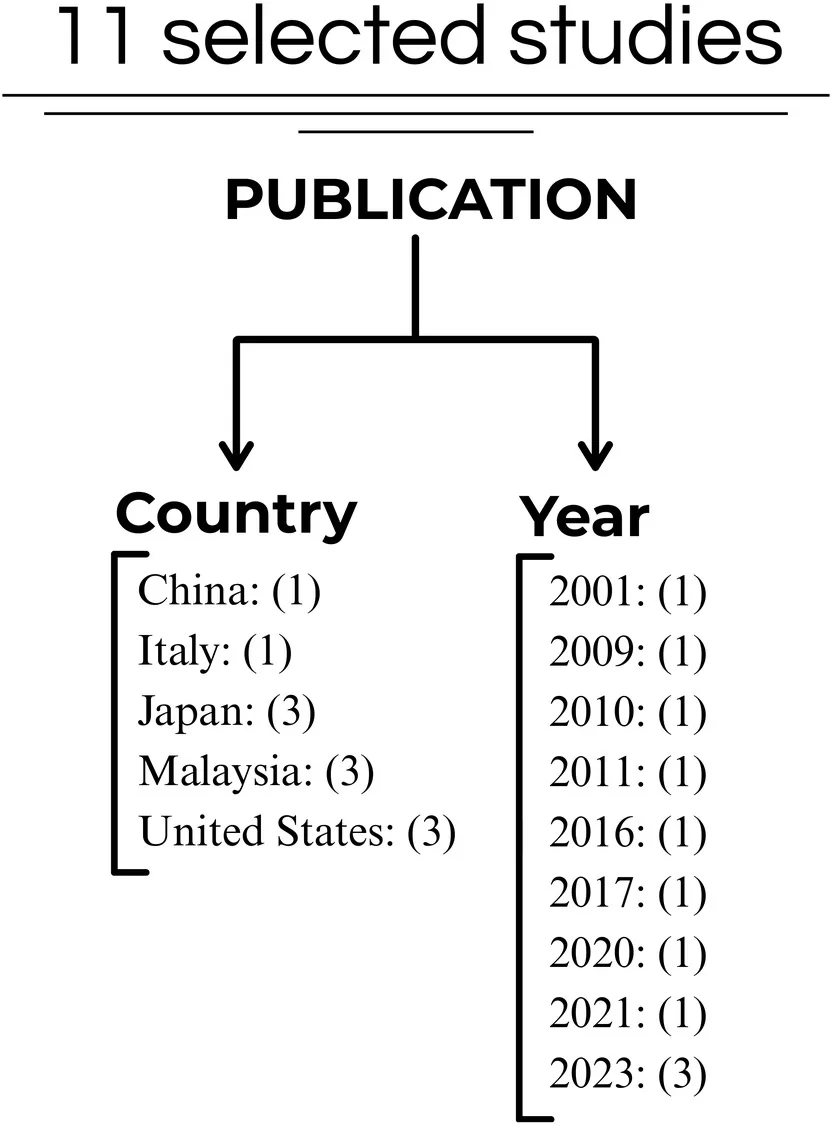
Correlation between publication countries and years of included articles. (Image created with Canva under the Canva Content License Agreement).

Finally, 11 studies met the eligibility criteria and were included in this review. The included studies were published between 2001 and 2023, with most originating from Japan, Malaysia, and the United States. These results are also described and illustrated in the figure below (Fig. 2).

The reliability assessment of the 11 included studies, conducted with the *in vitro* module of the ToxRTool, showed that five articles were classified as Category 1 (‘reliable without restriction’), while six were classified as Category 2 (‘reliable with restrictions’). No study received a Category 3 rating (‘not reliable’). The most consistent strengths across studies were the clear description of experimental methods, use of appropriate controls, and replication of assays. Conversely, the main shortcomings identified were the absence of randomization, lack of blinding, and limited reporting of biological replicates. The inter‐rater agreement between reviewers was high (Cronbach's α > 0.80), supporting the robustness of the scoring process.

### Study aims and main outcomes

The 11 studies included in this systematic review explored the effects of Lf in different experimental contexts related to wound healing. Most studies aimed to evaluate Lf's antimicrobial properties and its capacity to promote cellular processes critical for tissue repair, such as keratinocyte and fibroblast proliferation, angiogenesis, and ECM synthesis. Other studies focused on developing Lf‐based biomaterials, including hydrogels and dressings, designed to combine antimicrobial and pro‐regenerative activities. Sample sizes varied across studies, with most employing small‐scale *in vitro* assays (n = 3 to n = 6). Regarding the origin of Lf, both bLf and hLf were used, with bLf being the most frequently employed due to its accessibility and structural similarity to hLf. The main outcomes reported include enhanced cell proliferation and migration, increased collagen and hyaluronan production, and effective antibacterial activity against pathogens commonly associated with wound infections, such as *S. aureus* and *E. coli*. These findings are summarized in Table 1.

Although the initial search retrieved studies involving both *in vitro* and *in vivo* models, this review focused exclusively on *in vitro* assays to ensure greater consistency in experimental design and facilitate the analysis of Lf's cellular and molecular effects. *In vivo* studies presented significant heterogeneity in terms of animal species, wound induction methods, Lf dosage, and application protocols, making direct comparisons challenging. In contrast, in *vitro* models provided a controlled environment that allowed detailed investigation of fundamental processes such as keratinocyte migration, fibroblast proliferation, extracellular matrix deposition, and angiogenic signaling. However, the choice of cellular models in *in vitro* studies reflects a critical balance between experimental feasibility and physiological relevance [18, 19]. Immortalized cell lines, such as HaCaT keratinocytes and L929 fibroblasts, offer practicality and reproducibility but may exhibit altered gene expression profiles and reduced responsiveness compared to primary cells. On the other hand, primary keratinocytes, human embryonic kidney (HEK) cells and human dermal fibroblasts preserve native characteristics and provide more physiologically relevant responses, albeit with technical challenges and reduced reproducibility [20]. This variability in cellular models underscores the need for harmonized protocols to improve comparability across studies and strengthen the translational potential of *in vitro* findings.

### Cell models used

All included studies employed *in vitro* models to investigate the effects of Lf on key processes related to wound healing. Human keratinocytes and fibroblast cell lines were the most used cells, reflecting their central role in ECM synthesis, re‐epithelialization, and overall wound repair [21]. Interestingly, two studies employed alternative cell types: HEKs and L929 mouse fibroblasts [9, 22]. While HaCaT cells are widely used as an immortalized keratinocyte model for their robustness and ease of culture [23], HEKs represent a completely distinct human epithelial cell lineage of renal origin [22]. Therefore, explicit functional contrasts between these models must be interpreted with caution, as HEKs preserve native embryonic kidney epithelial behaviors rather than epidermal characteristics [24]. HaCaT cells tend to exhibit reduced responsiveness to pro‐inflammatory cytokines such as TNF‐α and IL‐1β, potentially underestimating the inflammatory phase of wound repair [25, 26]. Moreover, their differentiation capacity may differ from that of primary keratinocytes, particularly under stress or in response to bioactive molecules [27].

The use of L929 fibroblasts, a murine fibroblast line, is likely related to their widespread application in cytotoxicity assays and their ease of culture [28]. Though, species‐specific differences in signaling pathways and ECM production must be considered, as these cells may not fully recapitulate human dermal fibroblast behavior [29]. Such interspecies variability highlights the importance of selecting appropriate cellular models depending on the specific research objectives.

The use of HEKs thus allowed for a more physiologically relevant assessment of epidermal responses, while L929 cells were chosen for their extensive application in cytotoxicity assays and simplicity in maintenance [30, 31]. This variability in cellular models highlights potential differences in physiological relevance and may influence the comparability of findings across studies. In contrast, HEKs maintain a gene expression profile more reflective of physiological conditions [32]. They display a more accurate regulation of keratinocyte‐specific markers [33] and a natural responsiveness to inflammatory and oxidative stimuli [34]. These characteristics make HEKs highly valuable for studies aiming to elucidate molecular mechanisms in a context that closely mimics the native epidermis. However, their limited lifespan and sensitivity to culture conditions pose technical challenges and reduce their reproducibility across laboratories.

Most studies assessed cell viability using colorimetric assays, particularly AlamarBlue® and MTT, reflecting their widespread application for measuring cellular metabolic activity *in vitro* models. These assays are recognized for their sensitivity and suitability in detecting subtle cytotoxic effects, making them ideal for screening potential wound healing agents [35]. Exposure time and Lf concentrations varied across studies, but most reported no cytotoxic effects within the tested ranges. These methodological differences, however, contribute to variability in outcomes and highlight the need for standardized protocols in future research. Table 2 summarizes the experimental groups, assays employed to assess cytotoxicity, cell models used, and the key findings regarding cell viability in the included studies.

**Table 2:** Summary of cell viability assays in the included studies. This table provides an overview of experimental groups, cytotoxicity assays, cell types, and main findings related to cell viability. Cytotoxicity assay indicates the methods used to assess cell viability and metabolic activity (e.g., MTT, AlamarBlue®, LIVE/DEAD®). Cell type/lineage specifies the keratinocyte or fibroblast models employed in the *in vitro* experiments. Experimental approach describes the methodological strategies adopted in the studies, including dose–response designs, time‐course analyses, and other experimental considerations. Main outcomes summarize the key results regarding Lf's effects on cell proliferation, cytotoxicity, and overall biocompatibility.

References	Groups	Cytotoxicity assay	Cell type/lineage	Experimental approach	Main outcomes
Abdalla et al., 2020 [6]	Control; 16 μg/mL (Ag‐Lf); 32 μg/mL (Ag‐Lf); 62.5 μg/mL (Ag‐Lf); 125 μg/mL (Ag‐Lf).	AlamarBlue Assay	HDFs	Absorbance measurement by spectrophotometer reader	Reduction in cell viability observed from 16–125 μg/mL (24–72 h); non‐cytotoxic up to 24 h.
	Control; 32 μg/mL (Ag‐Lf); 62.5 μg/mL (Ag‐Lf); 125 μg/mL (Ag‐Lf).	LIVE/DEAD Cell Viability Assay		Fluorescence observation by fluorescence microscope	The control and cells treated with Ag‐Lf did not show the presence of dead cells.
Abdalla et al., 2021 [38]	Control; 32 μg/mL (Ag‐Lf); 62.5 μg/mL (Ag‐Lf); 125 μg/mL (Ag‐Lf).	AlamarBlue Assay	HDFs	Absorbance measurement by microplate spectrophotometer	Dose‐ and time‐dependent reduction in HDF viability (16–125 μg/mL, 24–72 h); hydrogels relatively non‐cytotoxic except at highest dose.
Fathil et al., 2023 [10]	Control; SL125*; SL250**; SL500***.	AlamarBlue Assay	HaCaT	Absorbance measurement by microplate spectrophotometer	SL125 non‐cytotoxic (24–72 h); higher doses (SL250/SL500) reduced viability at 48–72 h, indicating potential issues with prolonged exposure.
Tang et al., 2010 (a) [36]	(−) LF (+) LF [200 μg/mL]	Calcein‐AM/propidium iodide (PI) staining method	Primary human dermal fibroblasts	Fluorescence observation by fluorescence microscope	Holo‐rhLF (200 mg/mL) significantly increased fibroblast viability under starvation or TPA (66.5 → 81.2% and 55.3 → 73.2%).
		TUNEL assay			Holo‐rhLF reduced apoptosis from 34 → 1.7% (starvation) and 47 → 9% (TPA), protecting fibroblasts from cell death.
Tang et al., 2010 (b) [22]	(−) LF (+) LF [200 μg/mL]	Calcein‐AM/propidium iodide (PI) staining method	Normal human epidermal keratinocytes (HEKn) from neonatal foreskin	Fluorescence observation by fluorescence microscope	Holo‐rhLF (200 μg/mL) increased HEKn viability under starvation, TPA, and combined stress (58.9 → 88.9%, 66.7 → 90.6%, 47.2 → 77.3%) and reduced apoptosis.
		TUNEL assay			Holo‐rhLF (200 μg/mL) reduced HEKn apoptosis from 33.1 → 5.5% (starvation) and 34.8 → 2.8% (TPA), improving cell viability.
Yuan et al., 2021 [9]	BNC; BNC/LF; BNC/COL; BNC/LF/COL.	Calcein‐AM/PI	Mouse skin fibroblast cells (L929)	Fluorescence observation by fluorescence microscope	COL‐containing groups (BNC/COL, BNC/LF/COL) enhanced fibroblast attachment/proliferation; LF showed no cytotoxicity.
		Cell Counting Kit‐8 (CCK‐8) assay		Absorbance measurement using an enzyme‐linked immunosorbent assay reader	COL promoted fibroblast adhesion, normal morphology, and proliferation (BNC/COL, BNC/LF/COL); LF was noncytotoxic.

*Note: According to ISO 10993‐5 standard guidelines for the biological evaluation of medical devices, a material is only classified as cytotoxic if cell viability drops below 70%. In the studies by Abdalla et al. (2020, 2021), although a statistically significant decrease in metabolic activity was noted at higher concentrations compared to controls, cell viability consistently remained above this 70% safety threshold, justifying its classification as noncytotoxic*.

* SL125 = 125 μg/mL AgNPs + 250 μg/mL Lf + 0.015 μg/mL DsiRNA

** SL250 = 250 μg/mL AgNPs + 500 μg/mL Lf + 0.015 μg/mL DsiRNA

*** SL500 = 500 μg/mL AgNPs + 1000 μg/mL Lf + 0.015 μg/mL DsirRNA.

### Cell migration and proliferation

The effects of Lf on cell migration and proliferation were explored in the included studies using a variety of assays, primarily scratch wound healing assays, fluorescence microscopy, and colorimetric measurements (Table S1). These approaches enabled the assessment of two fundamental processes in wound repair: keratinocyte migration, which drives re‐epithelialization, and fibroblast proliferation, essential for granulation tissue formation and extracellular matrix (ECM) remodeling [21]. HaCaT keratinocytes and human dermal fibroblasts (NHDFs) were the most frequently used cell lines due to their relevance in these biological processes. Moreover, two studies employed alternative models, such as HEKs and L929 murine fibroblasts, to gain insights into more specific aspects of cell behavior.

### Pro‐healing effects of Lf

Across the studies, Lf demonstrated a consistent ability to enhance both keratinocyte migration and fibroblast proliferation. TANG and colleagues [22, 36] showed that recombinant hLf increased HaCaT migration and proliferation rates, which was associated with accelerated wound closure *in vitro*. Similarly, SAITO and colleagues [37] reported that bLf promoted fibroblast motility and ECM synthesis by stimulating hyaluronan and collagen production. In another study, ABDALLA and colleagues (2021) [38] demonstrated that mesoglycan‐Lf complexes exerted synergistic effects on keratinocyte migration, suggesting that such combinations may offer enhanced therapeutic benefits. The dose‐ and time‐dependent nature of these effects was highlighted in multiple studies, with higher concentrations of Lf generally producing stronger promigratory and proliferative responses without inducing cytotoxicity.

At the molecular level, Lf's prohealing effects are mediated through the modulation of key signaling pathways and the regulation of genes involved in tissue regeneration. Multiple studies highlighted the activation of the PI3K/Akt pathway, which promotes cell survival, proliferation, and migration by enhancing cytoskeletal dynamics and energy metabolism. For instance, RYU and colleagues [39] demonstrated that bLf interacts with the low‐density lipoprotein receptor‐related protein 1 (LRP1) on fibroblasts, activating PI3K/Akt signaling and upregulating tropoelastin (TE) expression. TE is a crucial ECM protein that contributes to skin elasticity and structural integrity.

Similarly, TANG et al. (2010) [22, 36] reported that recombinant hLf modulated keratinocyte migration and proliferation via MAPK and Rho/ROCK pathways. These pathways orchestrate cytoskeletal remodeling, focal adhesion turnover, and cell motility—essential processes for efficient re‐epithelialization. Moreover, TAKAYAMA et al. (2001) [40] identified increased phosphorylation of myosin light chain (MLC) mediated by Rho/ROCK and MLCK‐dependent pathways, further supporting Lf's role in enhancing fibroblast contractility and wound closure.

On the ECM level, SAITO et al. (2010) [37] demonstrated that bLf treatment significantly upregulated hyaluronan synthase 2 (HAS2) and type I collagen (COL1A1) in NHDFs, leading to enhanced ECM synthesis and fibroblast migration. These findings align with ABDALLA et al. (2021) [38], who observed synergistic effects on migration and ECM remodeling when Lf was combined with mesoglycan. Additionally, increased expression of vascular endothelial growth factor (VEGF) has been reported in some models, suggesting a contribution to angiogenesis in wound healing.

Overall, these studies highlight Lf's multifaceted molecular actions, including regulation of gene expression, modulation of ECM production, and activation of signaling pathways that coordinate cellular proliferation, migration, and angiogenesis. Integrating these mechanistic insights into a graphical model could help illustrate Lf's roles across the different phases of wound healing, from inflammation to tissue remodeling, and provide a conceptual framework for its therapeutic potential in regenerative medicine.

Lf acts as a central molecule modulating multiple intracellular signaling pathways involved in wound repair. Activation of the PI3K/Akt pathway promotes cell survival, proliferation, and migration. Upregulation of TE and phosphorylation of MLC, mediated by Rho/ROCK/MLCK pathways, enhances fibroblast contractility and wound closure. The MAPK/Rho‐ROCK axis regulates cytoskeletal remodeling, while induction of COL1A1 and HAS2 supports ECM synthesis and re‐epithelialization. These mechanisms collectively contribute to the inflammatory, proliferative, and remodeling phases of wound healing.

### Antimicrobial activity

**Fig. 3:**
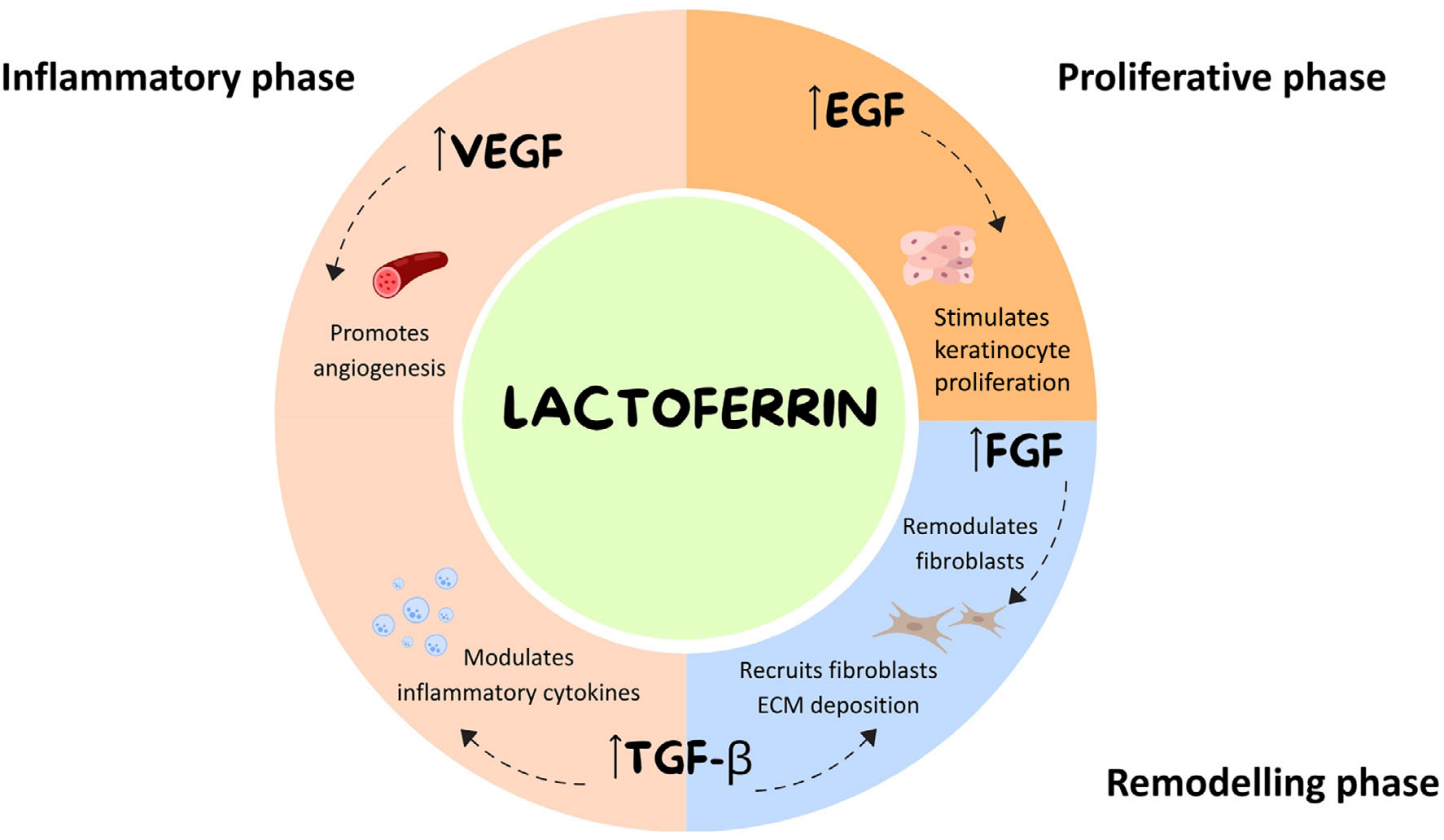
Modulation of growth factors by Lf in the wound microenvironment. Lf regulates the expression of key growth factors that orchestrate wound healing processes. VEGF stimulates angiogenesis; TGF‐β supports fibroblast recruitment and ECM deposition; EGF promotes keratinocyte proliferation and re‐epithelialization; and FGF contributes to fibroblast migration and tissue regeneration. Together, these paracrine effects highlight Lf's role in coordinating cellular responses and enhancing tissue repair.

In addition to its intracellular signaling effects, Lf also exerts paracrine actions by modulating the expression of key growth factors in the wound microenvironment. BELVEDERE and colleagues (2021) [7] have demonstrated that Lf upregulates VEGF, promoting angiogenesis, which is essential for supplying nutrients and oxygen to regenerating tissues. RYU et al. [39] reported that transforming growth factor‐beta (TGF‐β) contributes to fibroblast recruitment and ECM deposition, supporting granulation tissue formation. Moreover, ABDALLA et al. [38] and JIANG et al. [41] showed that Lf enhances the expression of epidermal growth factors (EGF) and fibroblast growth factors (FGFs), which stimulate keratinocyte proliferation, fibroblast migration, and tissue remodeling. These coordinated effects illustrate Lf's multifaceted role in orchestrating cellular responses and promoting efficient wound healing (Fig. 3).

VEGF stimulates angiogenesis, TGF‐β supports fibroblast recruitment and ECM deposition, EGF promotes keratinocyte proliferation and re‐epithelialization, and FGF contributes to fibroblast migration and tissue regeneration. Together, these paracrine effects highlight Lf's role in coordinating cellular responses and enhancing tissue repair.

In addition to cellular models, most studies evaluated the antimicrobial activity of Lf against *Staphylococcus aureus* and *Escherichia coli* (Table 3). These two species were prioritized because *S. aureus* is a predominant Gram‐positive pathogen frequently implicated in wound infections, while *E. coli* represents a Gram‐negative model organism commonly associated with contamination and delayed wound healing [42]. Their clinical relevance in chronic and acute wound environments underscores the importance of testing Lf efficacy against these bacteria.

**Table 3:** Summary of antimicrobial assays performed in the included studies. This table summarizes the antimicrobial assays employed to evaluate the effects of Lf and its formulations against clinically relevant microorganisms. The methods include agar diffusion, broth microdilution, and other standardized techniques to assess bacterial growth inhibition, with details on treatment groups and tested microbial species.

References	Groups	MOs tested	Antibacterial activity	Experimental approach	Main outcomes
Abdalla et al., 2020 [6]	Uncapped AgNPs; AgNPs; AgGO; Ag‐Lf; GO; Lf; Positive control; WESMS.	*S. aureus*; *Bacillus* sp.*;* *P. aeruginosa;* *E. coli*.	Agar well diffusion assay	Diameter of inhibition zone measurement by electronic digital Vernier caliper	AgNPs, Ag‐Lf, and AgGO showed strong antibacterial activity, outperforming the positive control and being especially effective against *P. aeruginosa*, while GO and Lf alone had no effect.
	Ag‐Lf; AgNPs; Uncapped AgNPs AgGO.		Microbroth dilution method	Microbial growth inhibition observation	AgNPs and Ag‐Lf inhibited Gram‐positive and Gram‐negative bacteria (MIC: 32 μg/mL), while AgGO showed higher MICs (125–250 μg/mL).
Abdalla et al., 2021 [38]	Gelatin hydrogel−Ag‐Lf (62.5 and 125 μg/mL); Blank gelatine hydrogel (−ve control); Ag‐Lf; Ciprofloxacin (+ve control).	*S. aureus*; *P. aeruginosa*.	Agar well diffusion assay	Inhibition zone measurement	Both bacteria demonstrated sensitivity to the hydrogel containing Ag‐Lf. Any inhibitory effect was not detected for the blank hydrogel (negative control). The inhibition of genipin cross‐linked gelatin hydrogel loaded with Ag‐Lf was lower than that of Ag‐Lf alone.
Fathil et al., 2023 [10]	AgLf; GC and SC (?)	*S. aureus;* *P. aeruginosa*; *E. coli*.	Microbroth dilution method	Microbial growth inhibition observation	Ag‐Lf showed antibacterial activity with a MIC of 125 μg/mL, while Lf alone (up to 1000 μg/mL) exhibited no effect against *E. coli*, *S. aureus*, or *P. aeruginosa*.
	Ciprofloxacin (20 μg/mL); CPX disk (5 μg); Lf (125, 250, 500 & 1000 μg/mL); AgLf (125/250 μg/mL); AgLf (1000/2000 μg/mL); SL gel containing AgLf (125/250 μg/mL); DL gel containing AgLf (125/250 μg/mL).		Agar well diffusion assay	Inhibition zone measurement	Ag‐Lf showed dose‐dependent antibacterial activity, with inhibition zones increasing up to 13.3 mm (*E. coli*), 11.3 mm (*S. aureus*), and 9.3 mm (*P. aeruginosa*) at 1000 μg/mL, while Lf alone (up to 1000 μg/mL) exhibited no antibacterial effect.
			Disk diffusion assay		
Yuan et al., 2021 [9]	Control; BNC; BNC/COL; BNC/LF; BNC/LF/COL.	*E. coli;* *S. aureus*.	Agar disk diffusion method	Inhibition zone measurement	LF‐impregnated BNC composites showed inhibition zones against *S. aureus* and *E. coli*, whereas pure BNC and BNC/COL had no antibacterial activity, confirming LF as the active component.
			Colony forming count (CFU) method	CFU counting	BNC/LF and BNC/LF/COL reduced *S. aureus* viability to 4% and 13.5%, and *E. coli* to 15.1% and 27.3%, with stronger inhibition against Gram‐positive bacteria likely due to charge interference.

Regarding antimicrobial assays, *S. aureus* and *E. coli* were the most frequently evaluated pathogens. This choice is consistent with their clinical relevance, as *S. aureus* is a leading Gram‐positive bacterium implicated in wound infections, while *E. coli* serves as a representative Gram‐negative organism often associated with contamination and delayed wound healing [42, 43]. Lf exhibits a multifaceted antimicrobial mechanism that contributes to its effectiveness against these pathogens [44]. Its iron‐binding capacity deprives bacteria of this essential micronutrient, exerting a bacteriostatic effect by limiting iron availability in the microenvironment [45]. Besides that, Lf directly interacts with bacterial cell membranes: in Gram‐negative bacteria such as *E. coli*, it binds to lipopolysaccharides (LPS), destabilizing the outer membrane and increasing permeability, which can ultimately lead to cell lysis against [46]. Gram‐positive bacteria such as *S. aureus*, Lf disrupts the cell wall by binding to teichoic acids and perturbing membrane integrity [47]. These interactions not only inhibit bacterial growth but also sensitize pathogens to other antimicrobial agents.

Another critical feature of Lf is its ability to inhibit biofilm formation, a common strategy employed by wound pathogens to evade host defenses and antimicrobial treatments [48]. Lf interferes with bacterial adhesion and quorum sensing pathways, reducing the establishment and maturation of biofilms on wound surfaces [49]. Beside these direct antimicrobial effects, Lf also modulates the host immune response in ways that support wound healing. It has been reported to enhance the recruitment and activation of neutrophils and macrophages, as well as promote the production of antimicrobial peptides such as defensins, and regulate the release of pro‐ and anti‐inflammatory cytokines [11, 50]. FATHIL et al. (2023) [10] highlighted that incorporating Lf into advanced biomaterials significantly reduces biofilm formation for pathogens such as *S. aureus* and *P. aeruginosa*, while supporting a microenvironment conducive to cell migration and balanced inflammation. The results regarding the analysis of inhibition of biofilm formation are shown in Table S2.

### Multifaceted role in wound healing

Despite methodological variations among studies, such as differences in Lf doses and treatment regimens, the majority reported consistent beneficial effects of Lf on wound healing outcomes. These combined properties suggest that Lf acts at multiple levels to reduce microbial load, modulate inflammation, and prevent infection‐related complications, positioning it as a promising adjunct in strategies for managing infected wounds and preventing chronicity.

In the early stages of repair, Lf's potent antimicrobial and anti‐inflammatory activities indirectly support healing by reducing bacterial colonization and modulating excessive inflammatory responses. These effects were particularly evident in the selected studies using *S. aureus* and *E. coli* as infection models, where Lf significantly reduced biofilm formation and enhanced antimicrobial peptide expression. Furthermore, as demonstrated by FATHIL et al. [10], Lf's immunomodulatory activity contributes to a more balanced inflammatory response, regulating the release of pro‐ and anti‐inflammatory cytokines to prevent excessive tissue damage while maintaining effective microbial clearance.

During the proliferative phase, Lf acts directly on tissue regeneration. Across the core studies, Lf consistently enhanced keratinocyte and fibroblast proliferation, processes essential for re‐epithelialization and ECM remodeling. Specifically, ADBALLA et al. (2020) [6] and JIANG et al. (2017) [41] showed that Lf enhances the expression of epidermal growth factors (EGF) and fibroblast growth factors (FGFs), which stimulate keratinocyte proliferation, fibroblast migration, and tissue remodeling. Concurrently, YUAN et al. [9] and FATHIL et al. [10] provided evidence that Lf—in both conjugated or unconjugated forms—contributes to wound repair by accelerating wound contraction and promoting angiogenesis, thereby improving vascularization and nutrient supply within the wound microenvironment.

In the final stages of remodeling, Lf supports the restoration of tissue structure. RYU et al. [39] observed increased collagen and hyaluronan synthesis, which are critical for restoring tensile strength. Additionally, they reported that transforming growth factor‐beta (TGF‐β) contributes to fibroblast recruitment and ECM deposition, supporting granulation tissue formation. Beyond these regenerative properties, the compound exhibited potent antioxidant activities, which are critical for preventing prolonged oxidative stress that could impair wound closure [50].

Despite these promising findings, the exclusive use of *in vitro* models among the 11 reviewed papers limits the direct extrapolation of results to complex *in vivo* systems. Furthermore, methodological heterogeneity and the lack of standardized dosing regimens complicate direct comparisons across the literature. Future research should aim to validate these findings in animal models and clinical trials and explore the incorporation of Lf into advanced wound dressings and biomaterials to maximize its therapeutic benefits.

## Concluding remarks

This systematic review highlights the multifunctional role of Lf in wound healing, emphasizing its relevance in modulating cellular processes, regulating signaling pathways, and integrating regenerative, immunomodulatory, and antimicrobial properties. By compiling diverse *in vitro* models and methodologies, the study provides a broad overview of Lf's potential applications while also identifying heterogeneities that should be addressed in future research. In this context, Lf emerges as a promising candidate for the development of innovative therapies in regenerative medicine and tissue engineering, reinforcing its importance in the bioprospecting of novel wound care strategies. Nevertheless, progress in this field relies on translational advances and well‐designed preclinical and clinical studies to validate and optimize its therapeutic applications.

## Conflict of interest

The authors declare no conflicts to declare.

## Author contributions

MLA, KCH, MSF, and GPC were involved in data curation, formal analysis, investigation, and software. MLA, KCH, MSF, GPC, and RGL were involved in methodology. RGL was involved in project administration and supervision. MLA and KCH were involved in validation and visualization. MLA was involved in writing—original draft. MLA and RGL were involved in writing—review & editing.

## Supporting information


**Table S1.** Summary of cell migration/proliferation assays in the included studies
**Table S2**. Summary of biofilm inhibition assays performed in the included studies.
