# Supplementary material for: Exploring the influence of lactoferrin on wound healing and related signaling pathways: a systematic review
Source: FEBS Open Bio. 2026 Sep 9:10.1002/2211-5463.70298. Online ahead of print. doi: 10.1002/2211-5463.70298 (PMC13555744; doi:10.1002/2211-5463.70298)
Supplement: Supplementary file 1 [file FEB4-9999-0-s001.pdf]

Table S1. Summary of cell migration/proliferation assays in the included studies. This table provides an overview of the experimental groups, wound healing assays, cell types, and main findings related to cell migration/proliferation. *Wound healing assay* indicates the methods used to assess cell migration and proliferation activity (e.g., scratch assay). *Cell type/lineage* specifies the keratinocyte or fibroblast models employed in the *in vitro* experiments. *Experimental approach* describes the methodological strategies adopted in the studies. *Main outcomes* summarize the key results regarding Lf's effects on cell migration and proliferation activity.

Reference	Groups	Wound healing assay	Cell type/lineage	Experimental approach	Main outcomes
Abdalla et al., 2020	Control;<br>32 µg/mL (Ag-LTF);<br>62.5 µg/mL (Ag-LTF).	Scratch assay	HDFs	Confocal microscope	Ag-LTF did not impair HDF migration, showing no adverse effects on cell motility during healing.
Abdalla et al., 2021	Control;<br>32 µg/mL (Ag-LTF);<br>62.5 µg/mL (Ag-LTF).	AlamarBlue® Assay<br>Scratch assay	HDFs	Confocal microscope	Ag-LTF-loaded gelatin hydrogel did not alter HDF migration, indicating low risk of adverse effects on cell motility during healing.
Belvedere et al., 2021	Control;<br>Mesoglycan;<br>Lactoferrin;<br>Mesoglycan + Lactoferrin.	Scratch assay	HaCaT, BJ and HUVEC	Time Lapse microscope	Mesoglycan and Lf individually enhanced cell migration in HaCaT, BJ, and HUVEC, while their combination showed a synergistic effect, producing the greatest migration distance among all groups.
		Cell invasiveness analysis		Optical microscope	In all cell lineages (HaCaT, BJ and HUVEC), the association of mesoglycan/Lf strongly potentiates the effects about the migration and invasion speed.
		Cell proliferation analysis	BJ	Hemocytometer cell counter	Mesoglycan and Lf individually enhanced BJ fibroblast proliferation, while their co-administration further increased proliferation rates from 72 h, showing a synergistic effect.
		Tube formation assay (*in vitro* angiogenesis)	HUVEC	Optical microscope (bright field images) and analysis of the number of branch points and tube lenght	The mesoglycan/Lf combination strongly enhanced *in vitro* angiogenesis, increasing branching points and tube length (~90%) versus all groups. Lf and mesoglycan alone also promoted angiogenesis, but with no difference between them.
		Cell differentiation markers analysis	HaCaT	Immunofluorescence and western blot analysis	**CK6 and CK10:** Mesoglycan/Lf co-treatment markedly increased the expression of CK6 and CK10 in HaCaT cells compared to single treatments.

			**E-cadherin:** E-cadherin expression was reduced by mesoglycan and lactoferrin individually, with a more pronounced decrease observed under combined treatment.<br><br>**ICAM-1:** Lf induced a stronger reduction in ICAM-1 levels than mesoglycan. The co-treatment further amplified this effect.<br><br>**Laminin-332 (LMN-332):** Mesoglycan promoted perinuclear expression of LMN-332, which increased and spread into the cytosol with Lf, and even more so under combined treatment.<br><br>**Integrin β1:** Integrin β1 expression was significantly enhanced by mesoglycan, moderately by Lf, and maximized under co-treatment.
Cell activation markers analysis	BJ	Immunofluorescence and western blot analysis*<br>(*WB only for vimentin, FAPα and Integrin β1)	**Vimentin**: The expression levels of vimentin did not undergone notable variation even if the protein appears better structured particularly in presence of the association of mesoglycan/Lf.<br><br>**N-cadherin**: The expression levels of N-cadherin was found with a significant decrease with mesoglycan/Lf and mainly in case of both compounds.<br><br>**FAPα**: BJ cells showed a notable increase of expression levels of FAPα when treated with sodium mesoglycan, with Lf and in a stronger way when these two substances have been administered together.

					**Integrin β1**: The expression levels of Integrin β1 gradually increased following mesoglycan, Lf and mesoglycan/Lf treatments, respectively.<br><br>**Vinculin**: A similar trend has been observed for vinculin, a cytoskeletal protein of the focal adhesion plaques whose expression augmented mostly in presence of the mixture (mesoglycan/Lf).
		Angiogenesis markers analysis	HUVEC	Immunofluorescence analysis	**VEGF**: In the presence of mesoglycan, of lactoferrin and strongly with both of them, HUVEC disclosed a gradual greater expression of VEGF.<br><br>**CD31**: The expressions levels of CD31 were largely increased when HUVEC cells have been treated with mesoglycan together with lactoferrin.<br><br>**N-cadherin**: In a very similar way founded on BJ cells, N-cadherin expression increased when lactoferrin has been added to mesoglycan.<br><br>**Fibronectin protein**: The results confirmed that fibronectin expression was strongly increased in presence of mesoglycan with lactoferrin. When mesoglycan and lactoferrin have been administered individually it has been only pointed out.<br><br>**Integrin β1**: The expression levels of Integrin β1 progressively increased in the experimental points mesoglycan, lactoferrin and mesoglycan/lactoferrin, respectively.
Fathil et al., 2023	**Low glucose:**<br>(Control, blank, AgNP, AgLTF,	Scratch assay	HaCaT	Inverted phase contrast microscope	In low glucose, Ag-LTF significantly enhanced HaCaT migration, achieving complete wound

	DL)<br><br>**High glucose:**<br>(Control, blank, AgNP, AgLTF, DL)				closure at 48 h, surpassing both the control and AgNPs alone. In high glucose, Ag-LTF showed only comparable migration rates to the control group
		Angiogenesis marker analysis		ELISA technique	Lf+DsiRNA complexed to AgNPs in hydrogel enhanced HaCaT migration and partially improved wound healing under hyperglycemic conditions, likely by increasing PGE2 levels and correcting PGT overexpression.
Jiang et al., 2016	CbLf;<br>SLs;<br>CbLf-SLs.	Cell proliferation markers analysis	HDFn	Quantitative real-time PCR	CbLf and CbLf-SLs assemblies enhanced HDFn proliferation by activating PI3K/Akt, PDGF, IGF1, and VEGF signaling pathways.
		Cell migration markers analysis			CbLf and CbLf-SLs assemblies promoted dermal fibroblast migration by upregulating genes encoding growth factors, ECM components, and related enzymes.
Ryu et al., 2017	Control;<br>Lf;<br>Apo-Lf;<br>Holo-Lf.	Cell elasticity markers analysis	Normal human dermal fibroblasts	Reverse transcription (RT)-PCR	Lf enhanced TE expression in fibroblasts via LRP1-mediated activation of PI3K/Akt1 signaling. TGF-β1 increased TE levels through PI3K/Akt2, highlighting distinct regulatory pathways.
Saito et al., 2011	Control;<br>BLF.	Cell proliferation and ECM (remodeling markers analysis)	NHDFs	Real-time PCR	bLf promoted hyaluronan and type I collagen synthesis in NHDFs by upregulating HAS2, HYAL2, and COL1A1 expression, enhancing ECM production in a dose-dependent manner.
				Western blotting	bLf dose-dependently enhanced HAS2 expression in fibroblasts, promoting hyaluronan synthesis through pathways distinct from those activated by PDGF-BB and TGF-β.

Takayama et al., 2001	Control;<br>Apo-bLf<br>Holo-bLf.	Collagen gel contract assay	WI-38 fibroblasts	Time Lapse microscope using a charge-coupled device camera.	bLf and hLf enhanced collagen gel contraction in a dose-dependent manner, independent of cell proliferation or iron-binding state.
		Scratch assay		Inverted phase contrast microscope	bLf enhanced WI-38 fibroblast motility and infiltration into the wounded area, supporting its role in promoting collagen gel contraction.
		Western blotting		Chemiluminescence system	Lf promoted fibroblast migration and collagen gel contraction via Rho/ROCK- and MLCK-dependent pathways, with MLC phosphorylation as a critical step. Src family PTKs and MEK/ERK cascades were also implicated.
Tang et al., 2009	**Cell proliferation:**<br>LF0;<br>LF10;<br>LF50;<br>LF100;<br>LF200.<br><br>**Cell proliferation (-/+FGF2):**<br>LF0-FGF2;<br>LF0+FGF2;<br>LF100-FGF2;<br>LF100+FGF2;<br>LF200-FGF2;<br>LF200+FGF2.<br><br>**Cell proliferation (-/+TGF-β1**<br>LF0–TGF-β1;<br>LF0+TGF-β1;<br>LF100–TGF-β1;<br>LF100+TGF-β1;<br>LF200–TGF-β1;	Cell proliferation assay	Primary human dermal fibroblasts	Vi-Cell analyzer (cell counting)	Holo-rhLf enhanced fibroblast proliferation in a dose- and time-dependent manner, showed synergistic effects with FGF2, and antagonized TGF-β1-mediated inhibition of cell growth.

	LF200+TGF-β1.				
	Control;<br>LF;<br>LF+PD98059;<br>FGF2;<br>FGF2+PD98059;<br>LF+FGF2;<br>LF+FGF2+PD98059.	Cell proliferation assay with MEK1 inhbitor PD98059			Holo-rhLf-induced fibroblast proliferation was blocked by PD98059, indicating MAPK pathway involvement.
	(+) LF;<br>(-) LF.	Cell attachment assay		Absorbance measurement by spectrophotometer	No significant difference was observed between cells treated with or without holo-rhLF.
	**Trans-well cell migration assay:**<br>LF0;<br>LF10;<br>LF50;<br>LF100;<br>LF200.<br><br>**Migration inhibition assay:**<br>Control;<br>LF;<br>LF+PD98059;<br>LF+PP1;<br>LF+Y27632.	Trans-well cell migration assay and migration inhibition assay		Phase-contrast microscope	Holo-rhLf strongly promoted fibroblast migration in a dose-dependent manner via MAPK/ERK, Src, and Rho/ROCK pathways.
Tang et al., 2010	LF0-Tf;<br>LF10-Tf;<br>LF100-TF;<br>LF0+Tf	Cell proliferation assay	HEKs	Vi-Cell analyzer (cell counting)	Holo-rhLf enhanced keratinocyte proliferation in a dose- and time-dependent manner, likely via MAPK pathway activation.

	LF0-Tf;<br>LF200-Tf;<br>LF200-Tf+PD98059	Cell proliferation assay with MEK1 inhbitor PD98059			Holo-rhLf enhanced keratinocyte proliferation, partially less potent than EGF, and this effect was blocked by PD98059, implicating MAPK pathway activation.
	**Cell scratch migration assay:**<br>LF0;<br>LF200;<br>LF200+PD98059;<br>LF200+PP1;<br>LF200+Y27632.<br><br>**Migration inhibition assay:**<br>Control shRNA;<br>Control shRNA+LF;<br>LRP1 shRNA+LF.	Cell scratch migration assay and migration inhibition assay		Phase-contrast microscope	Holo-rhLf enhanced keratinocyte migration via MAPK/ERK, Src, and Rho/ROCK pathways. LRP1 knockdown suppressed this effect, implicating LRP1-Lf interaction in cell motility.

Table S2. Summary of biofilm inhibition assays performed in the included studies. This table presents the assays used to investigate Lf's ability to inhibit biofilm formation or disrupt established biofilms. The methods include crystal violet staining, resazurin-based viability assays, and microscopy analyses, highlighting the tested bacterial strains, treatment conditions, and main outcomes.

Reference	Groups	MOs tested	Antibacterial activity	Experimental approach	Main outcomes
Abdalla et al., 2020	Uncapped AgNPs;<br>AgNPs;<br>AgGO;<br>Ag-LTF;<br>GO;<br>LTF;<br>Positive control;<br>WESMS.	*S. aureus*;<br>*P. aeruginosa.*	Biofilm formation inhibition by crystal violet assay	Absorbance measurement by microplate spectrophotometer and scanning electron microscopy	All nanocomposites exhibited anti-biofilm activity, and this was attributed to the ability of chitosan to alter the physical characteristics of bacterial cells and to the ability of Ag+ ions to interfere and regulate extracellular polymeric substances (EPS).
Abdalla et al., 2021	Gelatin hydrogel−Ag-LTF (62.5 and 125 µg/mL);<br>Blank gelatine hydrogel (−ve control);<br>Ag-LTF;<br>Ciprofloxacin (+ve control).	*S. aureus*;<br>*P. aeruginosa*.	Biofilm formation inhibition by crystal violet assay	Absorbance measurement by spectrophotometer	The anti-biofilm activity of hydrogels loaded with Ag-LTF had lower activity than that of Ag-LTF alone. This condition might be attributed by the gradual release of actives from the gelatin hydrogel and the size of Ag-LTF.
Belvedere et al., 2021	CTRL;<br>Meso;<br>Lf;<br>Meso+Lf.	*S. aureus*;<br>*P. aeruginosa*.	Biofilm formation inhibition by crystal violet assay	Absorbance measurement by spectrophotometer	For both strains, the action of mesoglycan remained at insignificant levels of effectiveness. The Lf alone can block for about the 82% and 78% the formation of *S. aureus* and *P. aeruginosa* biofilm, respectively. (90% and 87% compared to not treated *S. aureus* and *P. aeruginosa* bacteria, respectively).
Fathil et al., 2023	AgNPs;<br>LTF;<br>AgLTF.	*S. aureus*;<br>*P. aeruginosa*.	Biofilm formation inhibition by crystal violet assay	Absorbance measurement by spectrophotometer	Lf showed strong anti-biofilm activity against *S. aureus* and *P. aeruginosa*, with optimal effects at 250 µg/mL. DL hydrogels outperformed SL, likely due to slower Lf release (~49% vs. 86%), sustaining biofilm disruption over 24 h.
